# The effectiveness of pay-it-forward in addressing HPV vaccine delay and increasing uptake among 15–18-year-old adolescent girls compared to user-paid vaccination: a study protocol for a two-arm randomized controlled trial in China

**DOI:** 10.1186/s12889-022-14947-3

**Published:** 2023-01-07

**Authors:** Yifan Li, Chuanyu Qin, Shengyue Qiu, Yu He, Linchuan Pang, Xiaolan Xu, Vivian Wan-Cheong Yim, Shenglan Tang, Heng Du, Wenfeng Gong, Fan Yang, Joseph D. Tucker, Weiming Tang, Yun Wang, Leesa Lin, Mark Jit, Wei Song, Chunrong Li, Jennifer Smith, Jing Li, Dan Wu

**Affiliations:** 1 West China School of Public Health and West China Fourth Hospital, Sichuan University, Chengdu, China; 2 Yulin Community Health Service Center, Chengdu, China; 3 Department of Obstetrics & Gynaecology, Tuen Mun Hospital, Hong Kong, China; 4 Global Health Research Center, Duke Kunshan University, Jiangsu, China; 5 Duke Global Health Institute, Duke University, Durham, NC USA; 6 Bill and Melinda Gates Foundation, Beijing, China; 7 Institute of Population Research, Peking University, Beijing, China; 8 School of Medicine, University of North Carolina at Chapel Hill, Chapel Hill, NC USA; 9 Faculty of Infectious and Tropical Diseases, London School of Hygiene & Tropical Medicine, Room 360, Keppel St, London, WC1E 7HT UK; 10 SESH (Social Entrepreneurship to Spur Health) Team, Guangzhou, China; 11 University of North Carolina at Chapel Hill Project-China, Guangzhou, China; 12 School of Pharmacy, Chapman University, Orange, CA USA; 13 Department of Infectious Disease Epidemiology, London School of Hygiene & Tropical Medicine, London, UK; 14 Centre for Mathematical Modelling of Infectious Diseases, London School of Hygiene and Tropical Medicine, London, UK; 15 Maternal and child Health Department, Chengdu Health Commission, Chengdu, China; 16 Chengdu Women’s and Children’s Central Hospital, School of Medicine, University of Electronic Science and Technology of China, Chengdu, China; 17 Department of Epidemiology, UNC Gillings School of Global Public Health, Chapel Hill, NC USA

**Keywords:** Randomized controlled trial, Human papillomavirus (HPV), Vaccination, Vaccine hesitancy, Pay-it-forward (PIF), China

## Abstract

**Background:**

Human papillomavirus (HPV) vaccination could prevent cervical and other HPV-associated cancers attributable to vaccine-associated HPV types. However, HPV vaccination coverage among women aged 9–18 years old is low in China. Common barriers include poor financial affordability, minimal public engagement, and low confidence in domestically produced HPV vaccines. Pay-it-forward offers an individual a free or subsidized service then an opportunity to voluntarily donate and/or create a postcard message to support future people. This study aims to assess the effectiveness of pay-it-forward as compared to standard-of-care self-paid vaccination to improve HPV vaccine uptake among adolescent girls aged 15–18 years, who are left out in the current pilot free HPV vaccination task force in some parts of China.

**Methods:**

This is a two-arm randomized controlled trial in Chengdu, China. Eligible adolescent girls (via caregivers) will be randomly selected and recruited through four community health centers (one in the most developed urban areas, one in higher middle-income and one in lower middle-income suburban areas, and one in the least developed rural areas) using the resident registration list. A total of 320 participants will be randomized into two study arms (user-paid versus pay-it-forward vaccination) in a 1:1 ratio. The intervention assignment will be blinded to recruiters and participants using envelop concealment until the research assistants open the envelop to determine which treatment to deliver to each individual. The primary outcome of the study will be HPV vaccine uptake by administrative data. Secondary outcomes include costs, vaccine hesitancy, and the completion rates of the 3-dose HPV vaccination series.

**Discussion:**

This study will investigate an innovative pay-it-forward strategy’s effectiveness and economic costs to improve HPV vaccination among 15–18-year-old adolescent girls. Study findings will have implications for increasing HPV vaccine uptake in places where HPV vaccines are provided for a fee.

**Trial registration:**

Chinese Clinical Trial Registry (ChiCTR), ChiCTR2200055542. Registered on 11 January 2022.

**Graphical Abstract:**

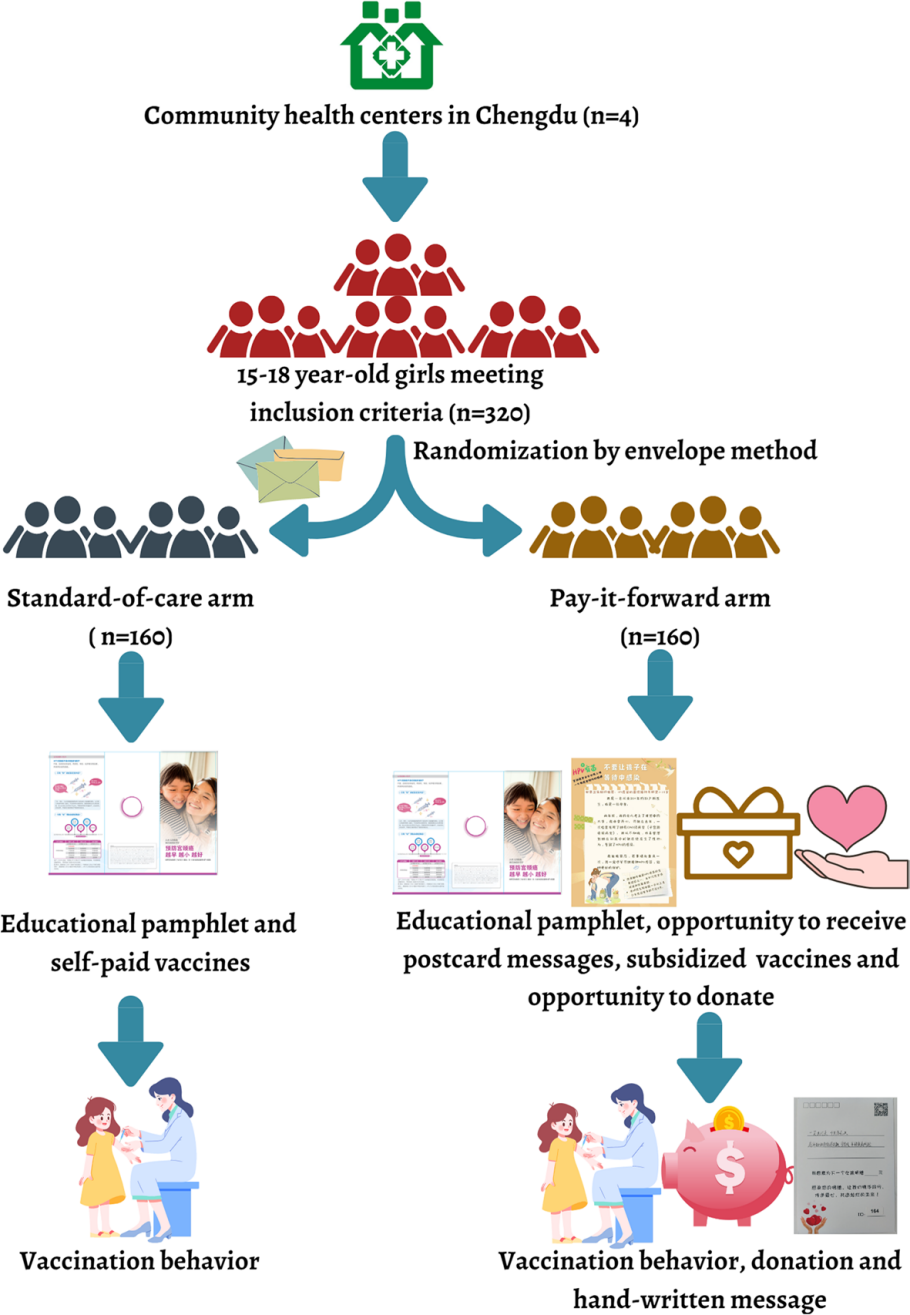

**Supplementary Information:**

The online version contains supplementary material available at 10.1186/s12889-022-14947-3.

## Background

Globally, about 341,831 people die from cervical cancer annually and over 15% of these deaths occurred in China in 2020 [1, 2]. An estimated 170 women die from cervical cancer each day in the country [1, 2]. Human papillomavirus (HPV) vaccination is a cost-effective preventive measure [2]. The Chinese Center for Drug Evaluation of National Medical Products Administration (NMPA) officially approved the imported bivalent vaccines (2v-HPV) in 2016, quadrivalent HPV vaccines (4v-HPV) in 2017 and the 9-valent HPV vaccine (9v-HPV) in 2018. Since 2019, NMPA approved two domestically produced 2v-HPV, Cecolin and Walrinvax, which is currently widely available in community-based health centers across the country. The domestic 2v-HPV vaccine is the least costly at 329 CNY (US$49.78 at the exchange rate of US$1 = 6.61CNY) per dose compared to the imported 2v-HPV at 600 CNY (US$90.79) per dose, while the 4v-HPV and 9v-HPV are much more expensive with a severe shortage of supply. However, a minority (< 1%) of 9–14 years old adolescent girls are vaccinated in 2019 [2], far behind the WHO 2030 goal of achieving 90% of girls fully vaccinated against HPV by 15 years of age. Among females aged 15–24 years in 2019, few (~ 11%) were vaccinated against HPV. Western China had the lowest vaccination rate at 8.6%, notably lower than in Central (10.6%) or Eastern China (13.7%) [3].

Low HPV vaccination coverage is related to poor availability and affordability, vaccine hesitancy [4], and inappropriate messaging [5]. HPV vaccines are generally not covered by insurances or government programs throughout China except in a few cities [6]. Guangdong Province, Fujian Province, and municipal Ordos and Chengdu provide subsidized HPV vaccination to girls aged 13–14 years old [7–9] and this might be one of the priorities in the country in the next decade. But this initiative leaves an important catch-up age group (e.g., 15–18 years) behind. The domestically produced 2v-HPV vaccine is cheaper and more available, but willingness to vaccinate remain low due to vaccine hesitancy [4, 10, 11]. Vaccine hesitancy, defined as the delay in acceptance or refusal of vaccination despite availability of services, is likely associated with minimal community engagement and inappropriate health messaging that focuses on promoting 4v- and 9v-HPV vaccines in the Chinese market. Innovative strategies are urgently needed to address the above issues and improve uptake of HPV vaccines available in the market among the 15–18-year age group.

**Fig. 1:**
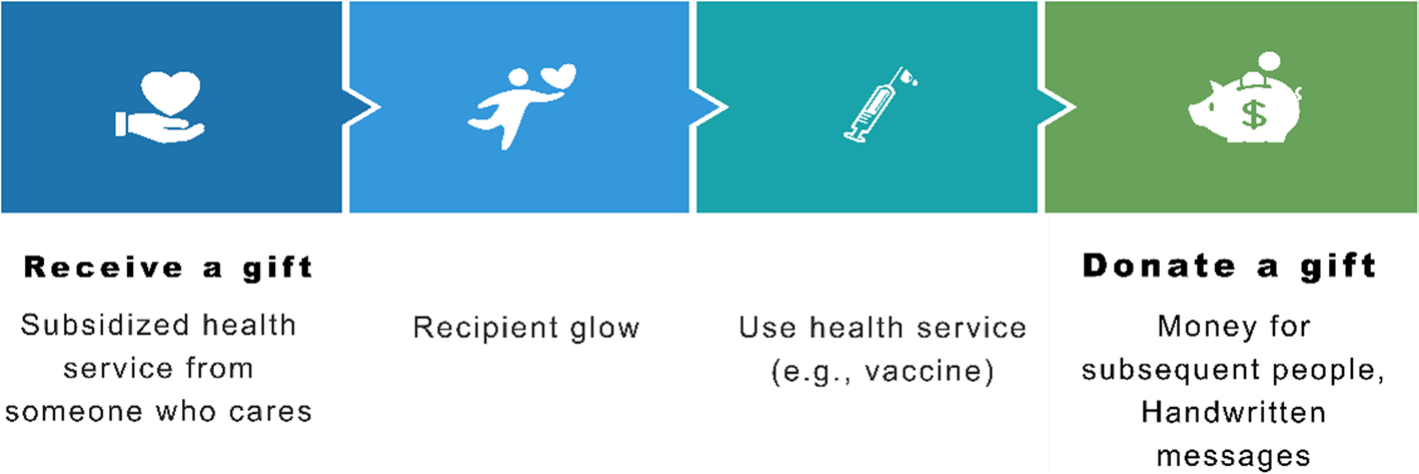
Pay-it-forward model overview. Pay-it-forward offers one individual an opportunity to receive subsidized HPV vaccination along with handwritten postcard messages, and a subsequent opportunity to voluntarily donate or create a postcard message to support more people

Pay-it-forward approach presents such an innovative strategy. Pay-it-forward offers someone an opportunity to receive subsidized HPV vaccination along with handwritten postcard messages, and a subsequent opportunity to donate and/or create a postcard message to encourage vaccination among their peers (Fig. 1) [12, 13]. Previous studies showed that the pay-it-forward model increased testing uptake of gonorrhea (56%) among gay men compared to the control arm (17%) and pay-it-forward participants (89%) who received a test donated to others [14]. A pay-it-forward intervention to improve influenza vaccine uptake among children and older people in China demonstrated notably higher vaccination rates (74%) compared to the standard-of-care arm (36%) [13]. It has also been shown to enhance public confidence in influenza vaccination compared to the standard-of-care arm. In essence, simple acts of kindness exemplified by pay-it-forward appears to be contagious [15] and hold promise to be leveraged to enhance public health services delivery for another vaccination initiative.

We describe here a two-arm randomized controlled trial (RCT) to evaluate the effectiveness of a pay-it-forward intervention to increase HPV vaccine uptake among adolescent girls aged 15–18 against the standard-of-care user-paid approach in four clinical sites of Western China. Pay-it-forward is hypothesized to be more effective than standard-of-care in increasing HPV vaccination among 15–18-year-old adolescent girls. Study findings will have implications for filling the gaps in HPV vaccine service delivery in places where HPV vaccines are provided for a fee and may serve as a transition model to free vaccine provision.

## Methods

### Trial design

**Fig. 2:**
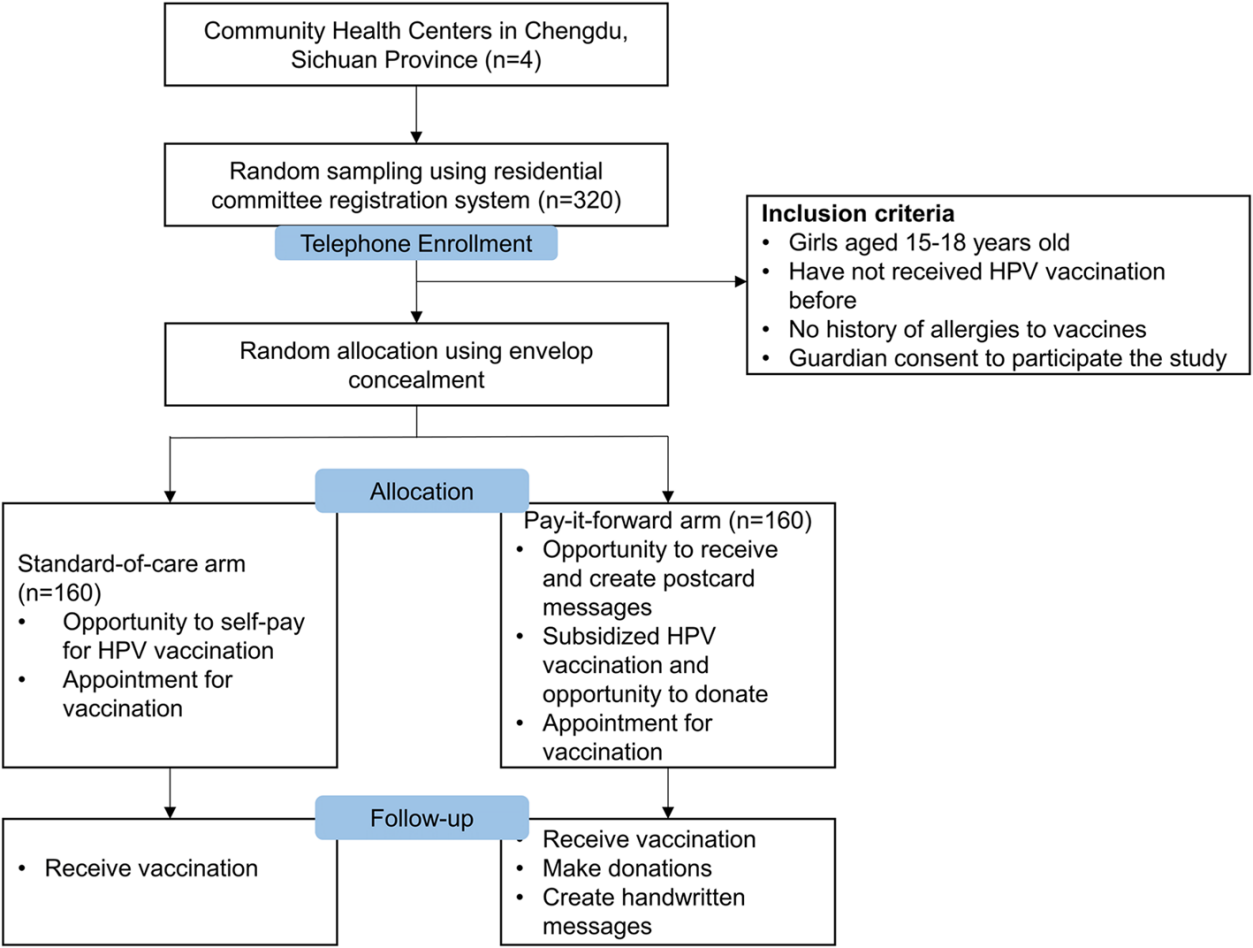
Overview of the RCT design

This is a two-arm randomized controlled trial among adolescent girls aged 15–18 at four study sites in Chengdu, Western China. Figure 2 shows the overview of the study. Eligible girls will be randomly selected from the residential committees’ registration system and invited to participate via caregivers. Residential committees are the community-led administrative organizations that help resolve essential livelihood issues and advocate for social welfare (e.g., public education, elderly care, essential health services) in the neighborhood. This community-based registration system intends to keep an electronic record of residents living in the neighborhood for 6 months or longer for management purpose [16] and is the smallest management unit in China. Consented participants will be randomly assigned using an envelope mechanism in a 1:1 ratio to the standard-of-care arm (user-paid vaccination at the market price) and the pay-it-forward arm (opportunity to receive postcard messages see Additional file 1: postcards, subsidized HPV vaccination, and donate or contribute a message).

### Study settings

Chengdu, with a population of over 21 million residents, is the capital city of Sichuan Province located in Western China [17]. Chengdu recently launched a government-supported initiative to subsidize HPV vaccination for girls aged 13–14 years old and reached a high uptake rate of over 90% among eligible girls within a short period of time [18]. This indicates that younger girls are also likely to benefit from the initiative in the future. But this excludes older age groups and there is a missed opportunity to promote HPV vaccination among the age group of 15–18 which is also the average age of the first sexual intercourse experience among Chinese youth [19, 20].

Four community health centers (one in the most developed urban areas, one in higher middle-income suburban area one in lower middle-income suburban areas, and one in least developed rural areas) are purposively selected based on the established infrastructure to provide HPV vaccination services, including a stable HPV vaccine supply and medical personnel designated for providing HPV vaccine services. Community health centers refer to primary care facilities providing essential medical care for common health conditions and preventive health services including vaccination to residents living in the neighborhood. Community health centers are the most common pathway for local people to receive vaccination in China and these organizations work closely with residential committees to provide essential public health services to residents. The four community health centers have agreed to coordinate resources for the study implementation. All research assistants, and community health staff involved in this project have been or will be trained using the same project training material (Additional file 2) and standard operational protocols (SOPs).

### Participants

The focus population of this trial are adolescent girls aged 15–18 years. The inclusion criteria are females: 1) aged 15–18 years; 2) living in the communities served by the selected community health centers; 3) with no HPV vaccination history, 4) with no prior allergies to vaccines; and 5) have a legal guardian who consents to participate the study. The exclusion criteria are females: 1) aged below 15 or above 18 years; 2) not living in the neighborhood served by the four community health centers; 3) received HPV vaccine(s); 4) ineligible to receive a vaccine based on clinical evaluation from a physician; or 5) a legal guardian refuses to consent to participate.

### Interventions

#### Pay-it-forward intervention arm

Participants assigned in this arm will receive the following 1) an educational pamphlet about cervical cancer, HPV vaccination, and differences in common types of HPV vaccines (Additional file 3); 2) information about the pay-it-forward vaccination program, including its purpose, the opportunity to receive postcard messages (Additional file 1 and details in the section of *Community engaged postcard messages*) and subsidized HPV vaccination, and the opportunity to voluntarily donate money towards someone else’s vaccine dose and write postcard messages. Participants will be told that the normal price to receive one dose of domestic 2v-HPV vaccine is RMB 329(US$ 49.78), and that previous participants donated money to cover the costs of the first dose of it along with postcard messages for them.

Participants (via caregivers) who agree to vaccinate can receive a vaccination on the same day of recruitment (when a girl is present) or make an appointment to visit again within the next two weeks for the administration of vaccination (when a girl is absent during the recruitment stage). After receiving a vaccination, girls, with caregivers or not, need to stay at the vaccination clinic for at least half-an-hour to observe any severe adverse events. During this period, research assistants will bring postcards and offer an opportunity to participants to donate and create handwritten messages for future participants. A donation/postcard collection box will be provided on-site, and a WeChat QR code will be provided to those who prefer an online donation to the program. WeChat is a multifunctional social mobile app embedded with monetary transaction functions. The donation will be anonymous, and project staff will be unaware of the donation amount. To link donations with other participant data, a postcard is assigned the same numerical ID of a participant and participants will be asked to put an intentional donation amount on the postcard along with their handwritten messages in the event of being willing to contribute. Neither the willingness to donate nor the amount of donation will affect their receiving the HPV vaccination services. The microdonations will be used to support future users. Donations and how these are used will be publicized periodically online via WeChat public accounts.

#### Standard-of-care arm

Participants assigned in this arm will be asked to pay out of pocket at the standard market price in the event of being willing to receive an HPV vaccination. The same educational pamphlet about cervical cancer, HPV vaccination, and types of HPV vaccines will be provided to participants in this arm, but they will not receive community-engaged messages or pay-it-forward information.

### Feasibility and acceptability pre-test

We conducted a feasibility pre-test with 100 eligible girls and field-tested the trial implementation process from January 4, 2022 to February 18, 2022 in the urban study site [21]. We collected survey data about caregiver attitudes towards options of HPV vaccines, vaccine hesitancy, and the pay-it forward intervention’s acceptability, appropriateness, and feasibility using adapted intervention measurements [22]. We organized a multi-stakeholder advisory meeting to discuss the pre-test results. These stakeholders were an HPV vaccination policymaker from Chengdu municipal health bureau, two public health practitioners in women and child health, two vaccine research experts, two global health researchers, and two infectious diseases researchers. We subsequently conducted four focus group discussions with a sub-set of participating caregivers (*n* = 21), and interviews with healthcare workers (n = 2) involved in the pre-test to better understand parental decision-making process for their daughter to receive HPV vaccination. The pre-test helped refine problem statement, sample size calculation, types of HPV vaccines to be provided in our trial, community-engaged postcard messages addressing knowledge gaps, and random sampling and recruitment strategies. These are described in detail below.

#### Problem statement

Our pre-test suggested that, due to the shortage of 4v and 9v-HPV vaccines in the market, the overwhelming majority of caregivers (91.5%) chose to delay their daughter’s HPV vaccination to wait for the above two types of vaccines to become available. This echoed a previous study [11] that there is greater demand for 4v and 9v-HPV vaccines than the currently available supply, but a delay or refusal of 2v-HPV vaccine uptake. A recent cohort study [23] demonstrated that 2v-HPV administered during the age of 12–13 years can reduce 87% of cervical cancer rates and 97% of grade 3 cervical intraepithelial neoplasia (CIN3), but such protection effects would significantly reduce for women receiving an vaccine at an older age, suggesting the importance of vaccination at an earlier age rather than later. The public health problem is therefore refined as how to address vaccine delay and increase *earlier* uptake of HPV vaccines that are currently available in the Chinese market among adolescent girls aged 15–18.

#### Sample size calculation

Our pre-test data showed that the pay-it-forward arm had an uptake rate of 98% (49/50) compared to 82% (41/50) in the standard-of-care arm. We, therefore, estimate a difference of 16% in vaccine uptake rates between the two arms. But because the current HPV vaccination rate among this population is generally low at around 10%, for the ongoing trial, we conservatively estimate an uptake rate of 20% in the standard-of-care arm and 36% in the pay-it-forward arm rather than the uptake rates in our pre-test. To achieve an 80% power to detect a difference between the two arms, 120 participants are needed respectively in the pay-it-forward and standard-of-care arms (Table 1). The test statistic used is the two-sided unpooled Z test and calculated by PASS 2021. The significance level of the test is 0.05. Given a non-response and/or dropout rate of 10%, 133 participants for each arm are needed. To allow sub-analyses on secondary outcomes and sub-group analyses, we will increase the sample size by 20% to 160 in each arm (40 from each clinic in each arm), leading to a total of 320 participants.

**Table 1:** Sample size calculation

	Sample size	Sample size (non-response and/or dropout rate of 10%)	ɑ	*β*
Pay-it forward arm	120	133		
Standard-of-care arm	120	133		
Total	240	266	0.05	0.20

#### Types of HPV vaccines

During the pre-test, we observed a substantial public demand for but an insufficient supply of 9v-HPV vaccine at the community health center. The center had to reserve the limited number of 9v-HPV vaccine for our pre-test participants, generating unintentional inequality of access to 9v-HPV vaccines between participants and not-participants. 4v-HPV vaccines were out-of-stock throughout the pre-test period. The advisory meeting revealed that the current supply of various types of HPV vaccines are insufficient to meet the demand in the next five to 10 years. Government-sponsored universal coverage of 4v and/or 9v-HPV vaccination for priority and catch-up groups (9–26 years) within the next decade in China is also unlikely. However, the supply of domestic 2v-HPV is relatively stable due to domestic pharmaceutical production lines. To ensure sufficient supply for all trial participants in the four study sites, we decided to use what is most available at the study sites (i.e., 2v-HPV vaccine). This may also help inform a cost-effective implementation strategy that complements future government-led schemes that use more accessible health products.

#### Community engaged postcard messages

We identified several key barriers to parental decision to vaccinate their girls against HPV. The level of awareness of HPV vaccines seemed high, but a complete understanding is lacking in terms of a) how effective of different types of HPV vaccines are in preventing cervical cancer, b) why it is optimal to vaccinate an eligible girl at an earlier age rather than later, and c) parental perception of age of sexual debut of their daughter. To integrate user perspectives in the designing process, we invited two university college students in preventive medicine to design three postcard cover messages. These cover messages were designed in response to caregiver preferences solicited during the focus group discussions, and focus on addressing the above knowledge gaps (Additional file 1). We will invite participating caregivers and girls to write on the back side of postcards to support other peers.

#### Random sampling and recruitment strategy

Our pre-test recruited participants through a WeChat newsletter announcing the study to local residents living in the neighborhood and who are served by the community health center. This recruitment strategy was used because it is how the community health center announces updates about vaccine services to the residents. However, by reflecting on the high uptake rate in the standard-of-care arm, we speculate that this may be largely attributable to a biased sample recruited online and these participants were already interested in HPV vaccination. These participants were economically better off (62% of the caregivers had an annual family income of 80,000RMB or above), and better educated (78% obtained university or higher-level education) compared to the local average levels. In addition, eligible participants who did not subscribe to the newsletter of the community health center do not have equal odds to be reached by our trial compared to those who subscribed.

We revised the sampling strategy to a simple random sampling using the residential committee registration system. Specifically, we will obtain a de-identified contact list of adolescent girls aged 15–18 years from the residential committee via community health center staff. This list includes all girls of this age range living in the neighborhood who also registered their names and contacts at the community health center to receive relevant health services there. We will then conduct the simple random sampling of eligible girls via generating and assigning a random digit to each individual using Microsoft Excel. These individuals will then be sorted by the random digits from the smallest to the largest, and we will select individuals from the top of the list for telephone recruitment until we reach the expected sample size at each study site.

The community health center will send a short text message via the patient management platform of the community health center to inform caregivers of selected participants about a follow-up telephone call from our project staff in next few days. The telephone recruitment will be conducted using landlines of the community health centers. Our project staff will introduce the general project during the telephone call and obtain verbal consent. Consented caregivers and/or participants will be invited to visit the community health center for enrollment. We aim to recruit 80 eligible participants at each study site.

### Randomization and treatment allocation

The research assistants will explain on-site the trial purpose, procedures, expected benefits and possible risks to participants, and obtain written consent to participate in the trial. Research assistants will then randomize participants into standard-of-care and pay-it-forward arms using a sealed envelope method. The envelope includes a random numerical ID generated using SPSS 21.0 that corresponds to the assigned arm (standard-of-care or pay-it-forward), a QR code for an online questionnaire survey, and postcard messages (pay-it-forward arm only). After receiving the allocated treatment, participants who agree to receive a vaccine will make an appointment through our trial and we will link them with a community health staff member who helps coordinate the administration of vaccination at the center.

We will use an information tracking sheet to record the assigned treatment, collect information about age (verified by identification card on-site), numerical ID and contact information. Personal information will be used for arranging clinical visits only and will not be accessible to staff who are not involved in the recruitment and clinical arrangement process.

### Participation and consent

Based on their assigned arm, participants will then be directed to research assistants who will deliver the allocated intervention. Participation in the study will be voluntary and anonymous. Access to other vaccination and medical services in the health center will not be affected in any way because of the participation in the study. Participants will be able to withdraw from the study at any time without giving any explanations.

All participants will be asked to complete a baseline survey after the consent. The questionnaires (Additional file 4) will collect information about their sociodemographic information, vaccine-related attitudes and behavior, pro-social intention and behavior, and their intended donation to support vaccination of other individuals (pay-it-forward arm only). We will add caregivers’ WeChat account for future follow-up and publicizing the microdonations and transparency.

### Blinding

In a behavioral intervention, both participants and people who deliver the treatment know which treatment they are receiving, and it is impossible to conduct a blind trial. But we will adopt several blinding techniques during the process to minimize biases. First, our trial will use an envelope concealment method. The process of generating the sequence of random digits and assembling envelopes is confidential to all on-site research assistants who recruit participants. Second, the allocated intervention will be concealed from study participants and research assistants onsite prior to assignment until they open the envelope. Second, the community health staff coordinator will not know which treatment a participant receives but only help coordinate within the clinic for administering the vaccination after the intervention delivery. Third, both the research assistants and/or community staff coordinator will help collect handwritten messages/donations, but they will be trained to maintain a high degree of consistency and neutrality in the process. Fourth, the allocated intervention (pay-it-forward or standard-of-care) will be blinded to physicians who prescribe an HPV vaccine throughout the process. Finally, the outcome assessors will be blinded from the knowledge of which intervention a participant receives.

### Data collection and management

Information about vaccine uptake (completion of one dose, two doses and all three doses respectively) and types of vaccines will be ascertained by clinical administrative data. Survey data will be collected using an online questionnaire. Administrative and survey data will be linked using numerical IDs. Cost data will be collected after we complete the trial. Dependent on the need of extra data for answering the proposed project objectives, participants can be further contacted via WeChat for additional follow- ups after ethical clearance.

### Outcome measures

#### Primary outcome

The primary outcome of the study will be first-dose HPV vaccine uptake in each arm after the intervention as assessed by administrative records. The choice of HPV vaccines will be dependent on the availability of vaccine products at the selected community health centers, primarily 2v-HPV vaccines.

#### Secondary outcomes

Secondary study outcomes include economic evaluation and costs indicators from the provider perspective, self-reported vaccine confidence and vaccine delay, and the completion rates of 2-dose and 3-dose HPV vaccination. Vaccine confidence, defined as public trust in the vaccine safety, importance and effectiveness [24], will be measured using survey items that have been adapted to the local context to assess vaccine confidence in China [25, 26]. Survey items are detailed in Additional file 4. Program costs will include personnel, facilities, equipment, materials and these will be categorized into start-up, fixed, and variable costs.

### Statistical analysis

#### Primary analysis

Descriptive analyses of socio-demographic and vaccination rates by study arms will be conducted. Intention-to-treat analysis will be conducted to compare vaccine uptake between the two arms. The primary analysis will evaluate the hypothesis that the pay-it-forward arm is more effective than the standard-of-care arm in increasing HPV vaccination. The uptake rates between the pay-it-forward and standard-of-care arms will be compared using logistic regression, reported as crude odds ratios (cOR) and adjusted odds ratios (aOR), and 95% confidence intervals (95% CIs), by adjusting for demographic backgrounds (e.g., education, income, employment status, sex of the guardian, and study sites).

#### Subgroup analysis

Effect modification analyses will evaluate whether the effect of the pay-it-forward intervention on HPV vaccine uptake vary in relation to the following factors: (1) family income levels; (2) level of sensitivity to vaccine costs (i.e., whether they perceive costs as a barrier to vaccination); (3) study sites; (4) sex of the guardian. For each factor of interest, three measures of association will be calculated: A) a crude measure of association between treatment group and vaccine uptake, B1) a measure of association between treatment group and vaccine uptake among all participants who report the factor of interest (e.g., lower family income, or high sensitivity to vaccination costs), B2) a measure of association between treatment group and vaccine uptake among all participants who deny a history of having the factor of interest (e.g., higher family income, or lower sensitivity to vaccination costs). Effect modification will be deemed present when B1 and B2 are different from one another, and at least one (B1 or B2) is different from A.

#### Secondary analysis

Markov model-based economic evaluation will be constructed to assess cost-effectiveness of pay-it-forward strategy in provider perspective. Cost for each program element will be collected, including personnel, materials, equipment and facilities, and micro-costing approach will be used to evaluate the costs of the standard-of-care and pay-it-forward arms. Pay-it-forward participant donations will be summarized and the fraction of the total cost of vaccination covered by pay-it-forward donation will be calculated. Mean donations by study site will be calculated and donation distribution by study site will be examined.

Vaccine hesitancy and vaccine confidence will be summarized using descriptive statistics and will be compared between the two arms. The completion rates of 2-dose and 3-dose HPV vaccination will be compared between the two arms. Comparisons between the 2 arms will be assessed with χ^2^ test, regression analyses, and 95% CIs will be calculated. All statistical tests will be two-sided, and statistical significance will be defined as *p* < 0.05.

### Missing data management

Loss-to-follow-up will be recorded during the six-month period after the enrollment of each participant. There may be missing data in self-reported secondary outcomes like vaccine hesitancy, but we do not anticipate any missing data in the primary outcome of vaccine uptake as this will be administrative data. If an outcome is missing for less than 15% of participants, analyses will use a complete-case approach. If an outcome is missing for 15% of participants or above, we will use multiple imputation to estimate the primary outcomes.

## Discussion

China has a substantial cervical cancer burden. Women in Western China have higher HPV infection prevalence than those in Central and Eastern areas [27] but have lower vaccine uptake. The main reasons for low HPV vaccination uptake are unavailability and unaffordability, vaccine hesitancy, and lack of appropriate health messaging. Some local governments are starting to support HPV vaccination among girls aged 13–14 years through subsidizing vaccination costs, but this leaves other important catch-up age groups (e.g., 15–18 years) behind. In addition, there are limited community-engaging programs and interventions to decrease vaccine hesitancy and increase HPV vaccine uptake in China. Innovative strategies are needed. Our pay-it-forward strategy provides a unique opportunity to investigate ways to address the above issues. This RCT attempts to evaluate the effectiveness and costs of pay-it-forward against routine practices in addressing vaccine hesitancy and increasing timely uptake of HPV vaccines available in the market among girls aged 15–18 by tapping into community generosity and pro-social interventions.

Our trial findings can have important implications for research, practice and policy. This is a rare community-engaged intervention to increase HPV vaccine uptake among underserved populations in a middle-income country. The research findings may have important implications for pro-social interventions to address vaccine hesitancy, and other common factors influencing vaccination behaviors and increasing uptake domestically and globally. Then the trial implementation process can demonstrate to some extent the adaptability of a pay-it-forward model in vaccine services and inform evidence-based practice in future vaccine programs. Third, while local governments may have limited finances for many competing health priorities and be unable to cover all priority groups for HPV vaccination at the same time, our pay-it-forward may serve as a transition model to help cover catch-up age groups and lower government pressure by mobilizing multiple players in the healthcare system (e.g., end-users, community-based healthcare providers, donors, pharmaceutical companies etc). Finally, given that there is an unstable supply in the Chinese context, we will not include 4v and 9v-HPV vaccines as an option for participants to choose. This may somewhat limit our understanding of participants’ uptake of 4v and 9v-HPV vaccines, but because imported 9v-HPV vaccines are unaffordable for many, the likelihood of the imported 9v-HPV being included in government supported universal health programs is low in the next decade. Domestic 9v-HPV vaccines are still in Phase III clinical trial and production capacity for 9v-HPV vaccines in the next five to 10 years in the country is also limited. Therefore, our study focuses on HPV vaccine products that are most available in the market will have important implications for evidence-based policymaking as well as effective public messaging to improve timely HPV vaccine uptake.

## Supplementary Information

**Additional file 1.** 

**Additional file 2.**

**Additional file 3.** 

**Additional file 4.** 

## Data Availability

Data sharing is not applicable to this article as no datasets were generated or analysed during the current study.

## Abbreviations

HPV: Human papillomavirus
CFDA: China Food and Drug Administrative
2v-HPV: Bivalent HPV
4v-HPV: Quadrivalent HPV
9v-HPV: 9-valent HPV
RCT: Randomized controlled trial
SOPs: Standard operational protocols
cOR: Crude odds ratios
aOR: Adjusted odds ratios
CIs: Confidence intervals
