# Supplementary material for: The effectiveness of pay-it-forward in addressing HPV vaccine delay and increasing uptake among 15–18-year-old adolescent girls compared to user-paid vaccination: a study protocol for a two-arm randomized controlled trial in China
Source: BMC Public Health. 2023 Jan 7;23:48. doi: 10.1186/s12889-022-14947-3 (PMC9824916; doi:10.1186/s12889-022-14947-3)
Supplement: Supplementary file 1 — Additional file 1. [file 12889_2022_14947_MOESM1_ESM.pdf]

HPV疫苗

Do NOT let your child get infected with HPV while waiting for your preferred choice of HPV vaccine

Cervical cancer is a vaccine-preventable disease.

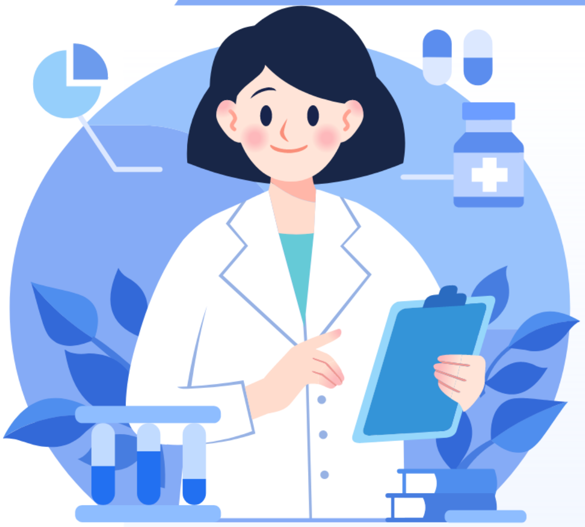

In the Chinese population, 84.5% of cervical cancer cases are caused by two types of HPV (16 and 18). 2-valent or 4-valent vaccines can potentially prevent 85% of cancers, while 9-valent vaccines can prevent 91%

HPV vaccines and the HPV types	protected against
Bi-valent	HPV16、18
4vHPV	HPV16、18、6、11
9vHPV	HPV16、18、6、11、31、33、45、52、58

Earlier vaccination is more important than a 4vHPV/9vHPV.

HPV疫苗

Cervical cancer is one of the few cancers that is almost totally preventable via vaccination

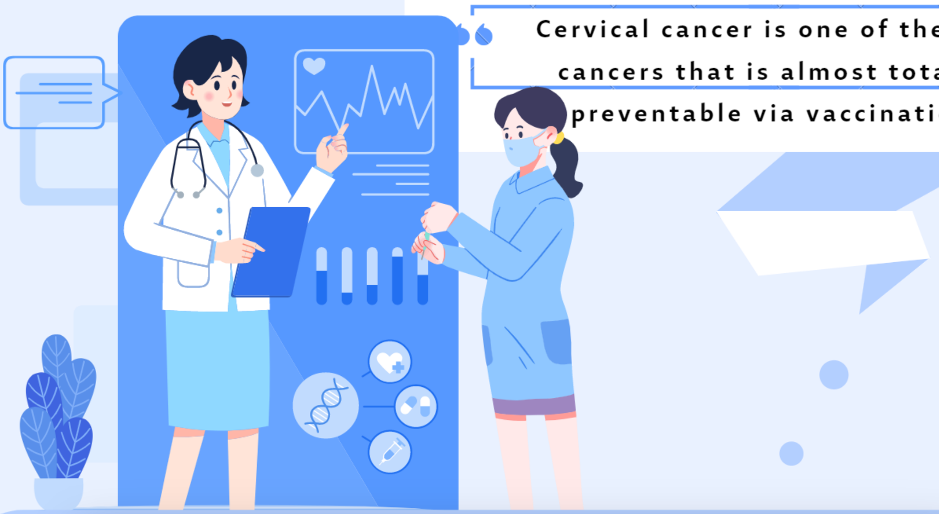

The earlier, the younger, the better

A British cohort study found that the protection against cervical cancer increases with earlier age of HPV vaccination.

Age of vaccine	12-13	14-16	16-18
The incidence reduced by	87%	62%	34%

- Evidence suggests the earlier the vaccination age, the better the effectiveness. Get whatever HPV vaccines are available in the market.

Experts suggest: do not miss the best vaccination age window because of waiting for a preferable vaccine.

HPV疫苗

Cervical cancer is one of the few cancers that is almost totally preventable via vaccination

Do NOT let your child get infected with HPV while waiting for your preferred HPV vaccine

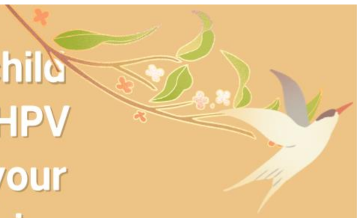

WHO recommends: the optimal age range for HPV vaccination is 9-14 years old.

I am an obstetrician and gynecologist with over 20 years experiences and also a mother of a young girl.

2 years ago, my daughter was admitted to her dream university, and I was thrilled. But last year, she was diagnosed with CIN2 (a precancerous condition of the cervix). I never knew or expected that she had started to have sex in high school until now.

I regretted not knowing about this earlier. If I had a chance to do this all over again, I would have taken her to get HPV vaccination as early as possible and give her the best protection against HPV infection.

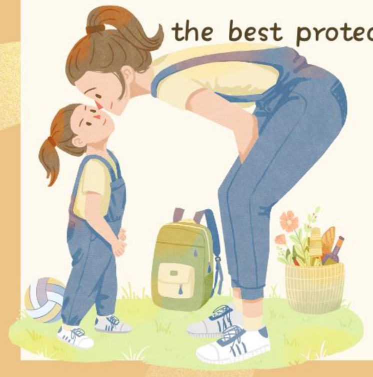

- Sexual contact is the primary transmission route of HPV infection.
- Evidence showed that, among Chinese adolescents who had sex, the average age of sexual debut was < 18 years.
