# Supplementary material for: The effectiveness of pay-it-forward in addressing HPV vaccine delay and increasing uptake among 15–18-year-old adolescent girls compared to user-paid vaccination: a study protocol for a two-arm randomized controlled trial in China
Source: BMC Public Health. 2023 Jan 7;23:48. doi: 10.1186/s12889-022-14947-3 (PMC9824916; doi:10.1186/s12889-022-14947-3)
Supplement: Supplementary file 2 — Additional file 2. [file 12889_2022_14947_MOESM2_ESM.pdf]

HPV“接力种”项目现场工作介绍

2022年

现场流程图

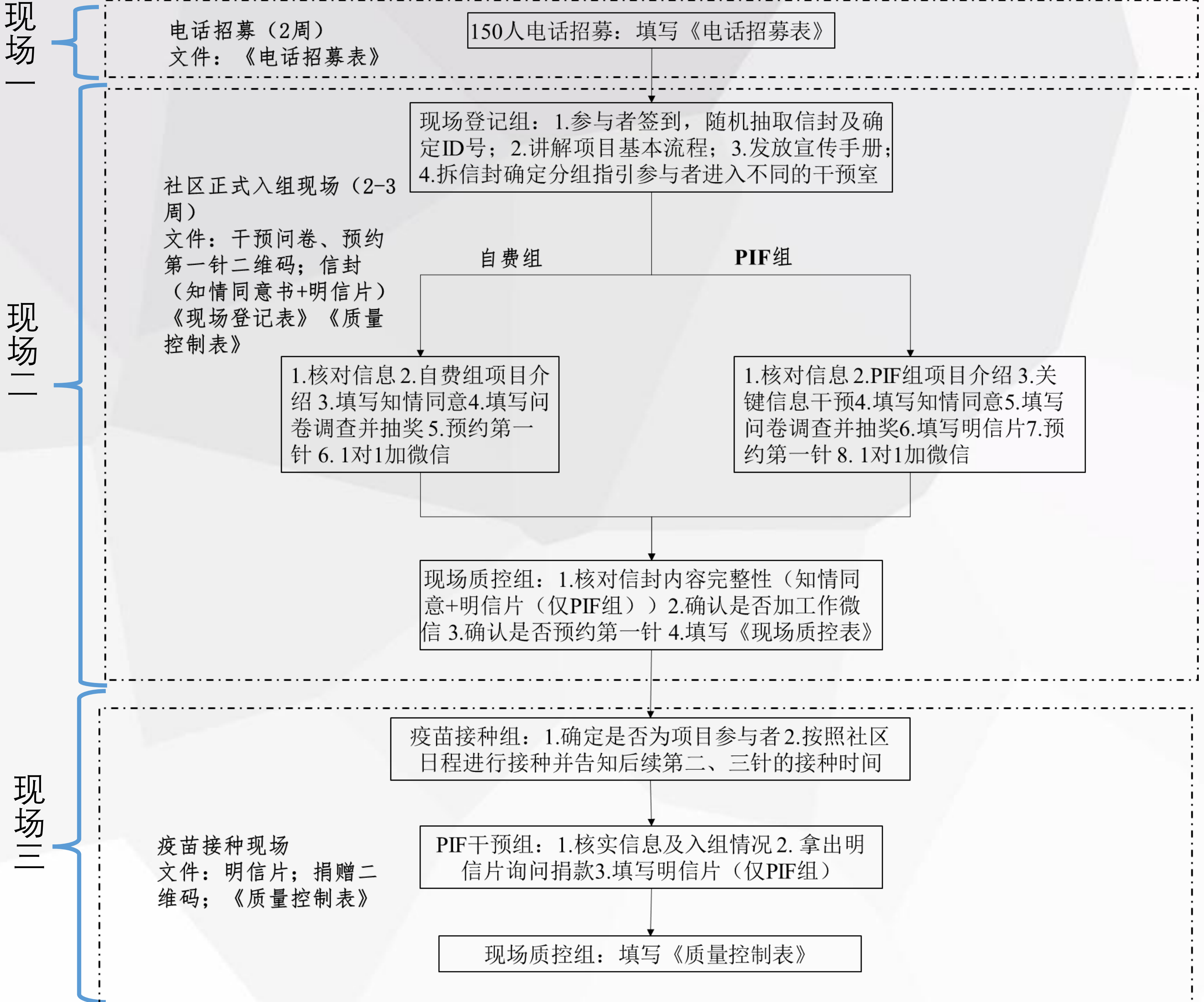
现场一
电话招募（2周）
文件：《电话招募表》
150人电话招募：填写《电话招募表》
现场二
社区正式入组现场（2-3周）
文件：干预问卷、预约第一针二维码；信封（知情同意书+明信片）《现场登记表》《质量控制表》
现场登记组：1.参与者签到，随机抽取信封及确定ID号；2.讲解项目基本流程；3.发放宣传手册；4.拆信封确定分组指引参与者进入不同的干预室
自费组
PIF组
1.核对信息 2.自费组项目介绍 3.填写知情同意4.填写问卷调查并抽奖 5.预约第一针 6. 1对1加微信
1.核对信息 2.PIF组项目介绍 3.关键信息干预4.填写知情同意5.填写问卷调查并抽奖6.填写明信片7.预约第一针 8. 1对1加微信
现场质控组：1.核对信封内容完整性（知情同意+明信片（仅PIF组））2.确认是否加工作微信 3.确认是否预约第一针 4.填写《现场质控表》
现场三
疫苗接种现场
文件：明信片；捐赠二维码；《质量控制表》
疫苗接种组：1.确定是否为项目参与者 2.按照社区日程进行接种并告知后续第二、三针的接种时间
PIF干预组：1.核实信息及入组情况 2. 拿出明信片询问捐款3.填写明信片（仅PIF组）
现场质控组：填写《质量控制表》

PART 1

电话招募

电话招募

3名经统一培训的工经作人员进行电话招募，若其满足以下条件即可邀请参加现场活动：

①女孩年龄在15-18周岁

②女孩未接种过HPV疫苗

③父母有兴趣参加这项活动

文件：《电话招募表》

PART 2

社区入组现场

01 健康干预（干预前问卷）

02 信封抽取+问卷调查（干预后）

03 质量控制

①健康干预

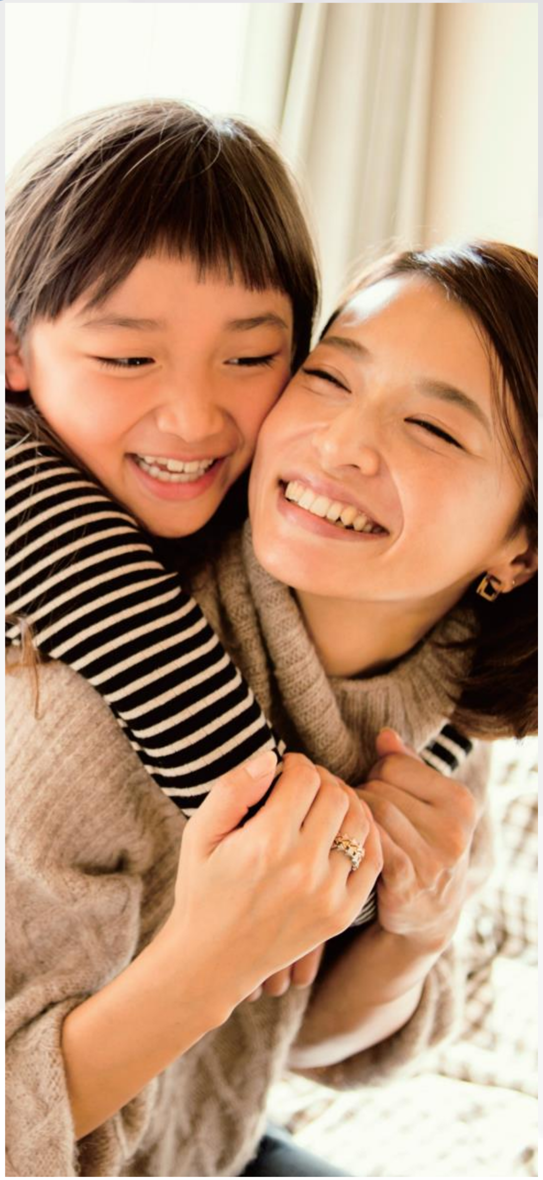

- 登记组：签到；发放宣传册；随机抽取信封袋并确定ID号；
- 工作人员介绍PIF项目
- 以小组为单位组织宣传册阅读
- 一天两组，分别为上下午，每组10人左右。

所需材料：

宫颈癌及HPV疫苗宣传手册

《现场招募登记表》

②信封抽取+问卷调查

文件:
1.信封（知情同意+明信片）
2.《现场质控表》
3.干预后问卷二维码
4.第一针预约二维码
5.工作微信二维码

若不预约，拒绝接种，请在《现场质控表》内填写原因。

随机抽取信封

↓

由登记组人员拆信封并确定分组

↓

据组别进入不同问卷调查室

↓

1.知情同意书
2.干预后问卷（问卷星）
3.干预后问卷结束后进行抽奖
4.预约第一针时间
5.询问捐款意愿（仅PIF组）

↓

1对1加工作微信并质控

↓

结束：感谢配合

③质量控制

附件 6：质量控制表

入组现场质量控制表

20____年____月____日

ID 号	姓名	1 对 1 加微信	分组	是否预约	接种类型				档案袋		拒绝原因	质控员	是否捐款	金额	备注
					2v 国产	2v 进口	4v 疫苗	9v 疫苗	知情同意	明信片（仅 PIF）					

文件：入组现场质控表

设备：签字笔

• [illegible]

• 明信片（仅PIF组）

3. 确认是否在小程序上预约接种时间

4. 确认是否一对一加微信

5. 填写《现场质控表》

PART 3

疫苗接种现场

01 接种组

02 问卷调查组

03 质量控制组

疫苗接种现场

- 工作地点：社区医院
- 文件：接种[illegible]
现场工作流程[illegible]
- 设备：签字[illegible]
爱心[illegible]
爱心卡片；
透明募捐箱

接种组

确认参与者身份，指引参与者进入问卷组

接[illegible]

附件 6：质量控制表

入组现场质量控制表

20____年____月____日

ID 号	姓名	1 对 1 加微信	分组	是否预约	接种类型				档案袋		拒绝原因	质控员	是否捐款	金额	备注
					2v 国产	2v 进口	4v 疫苗	9v 疫苗	知情同意	明信片（仅 PIF）					

质控组

核实参与者信息、
核实资料完整情况
填写《接种现场质控表》

THANKS!
