# Supplementary material for: The effectiveness of pay-it-forward in addressing HPV vaccine delay and increasing uptake among 15–18-year-old adolescent girls compared to user-paid vaccination: a study protocol for a two-arm randomized controlled trial in China
Source: BMC Public Health. 2023 Jan 7;23:48. doi: 10.1186/s12889-022-14947-3 (PMC9824916; doi:10.1186/s12889-022-14947-3)
Supplement: Supplementary file 3 — Additional file 3. [file 12889_2022_14947_MOESM3_ESM.pdf]

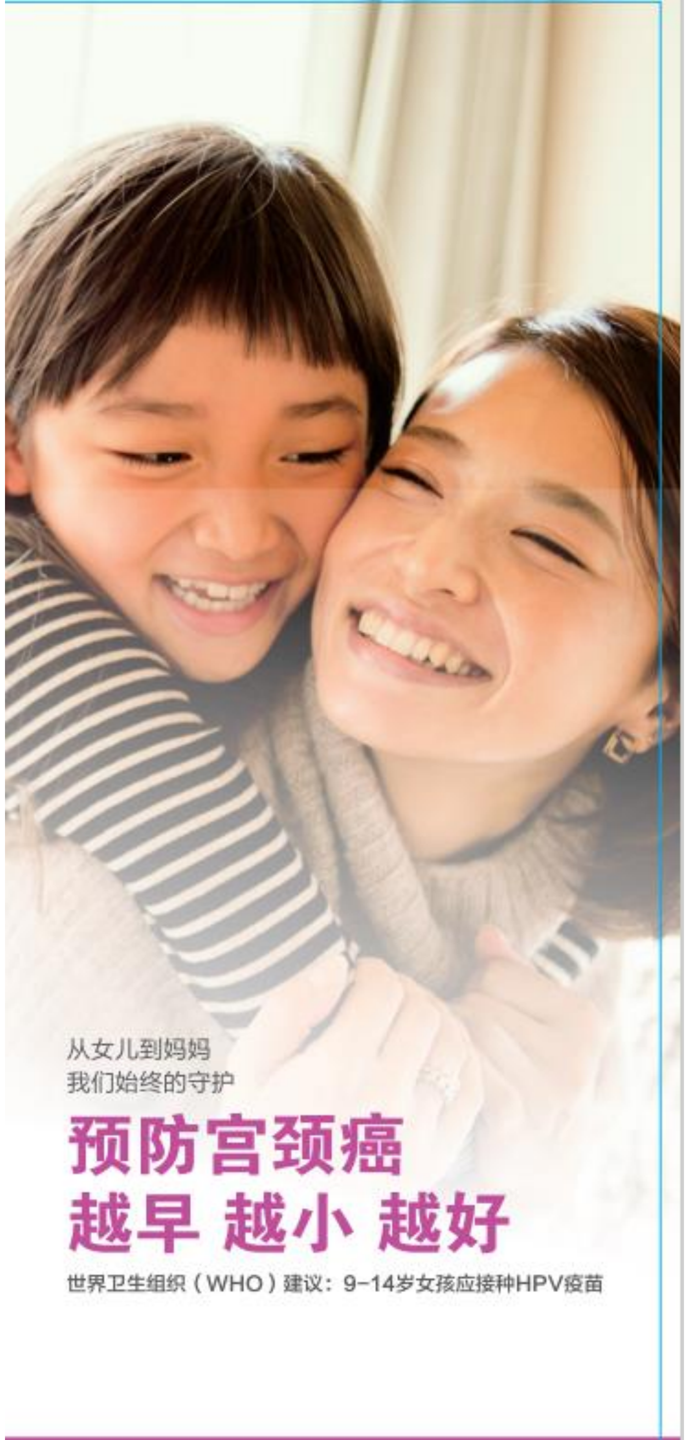

We protect daughters and mothers

Cervical cancer prevention
The earlier, the younger, the better

WHO recommends primary target population for HPV vaccination is adolescent girls aged 9–14 years for preventing cervical cancer

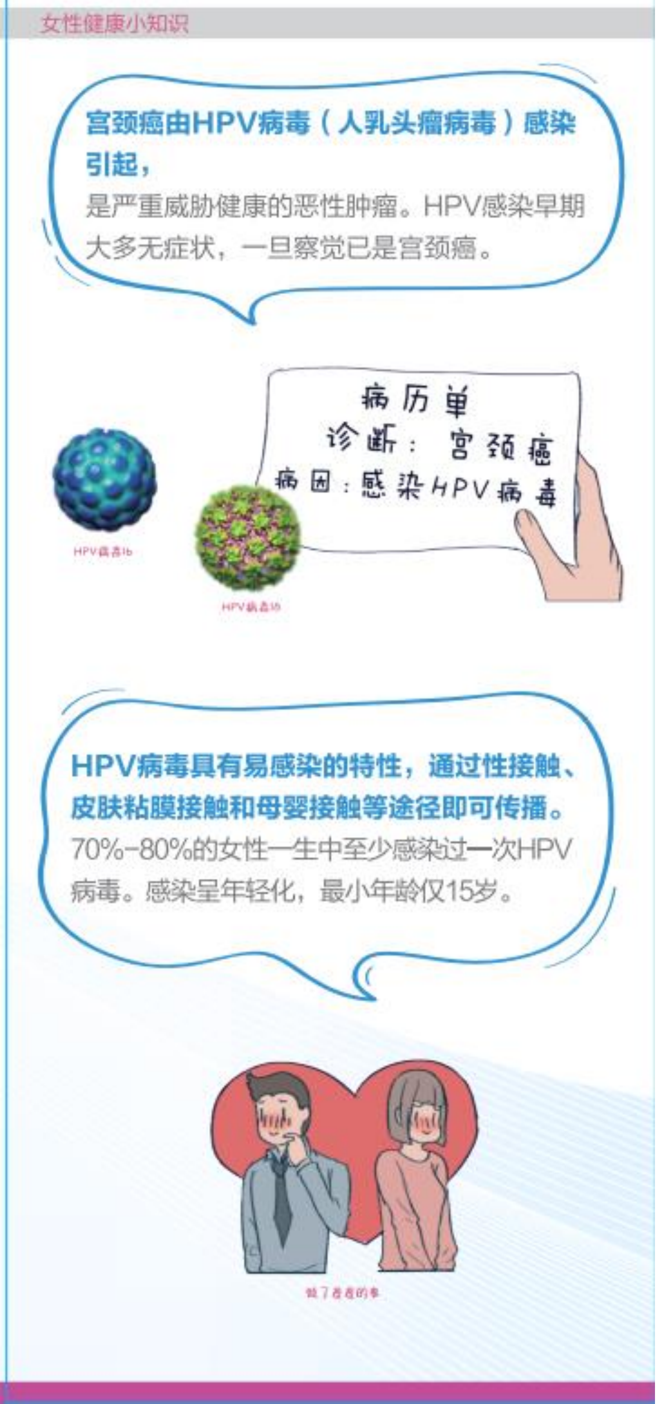

Persistent infection with high-risk HPV types can lead to cervical cancer, which is a serious threat to health. HPV infections are mostly asymptomatic in the early stage, but if left undetected, there is a high risk of progression.

HPV viruses are spread through contact with infected genital skin, mucous membranes, or bodily fluids, and can be transmitted through sexual intercourse and vertical transmission of HPV from mother to child.

About 70-80% of women have been infected with HPV at least once in their lives. Infection tends to occur at a younger age, with the youngest being 15 years old.

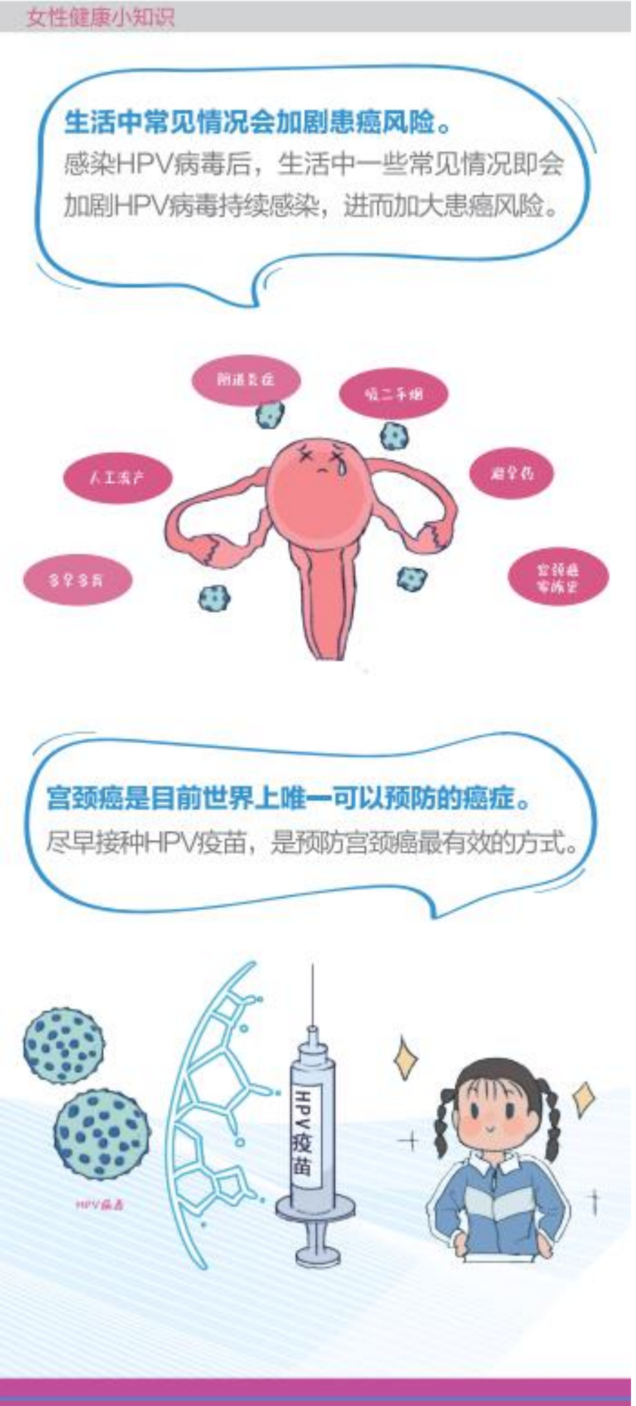

After acquiring HPV viruses, common factors in daily life may become risk factor for persistent infection with high-risk HPV types, contributing to progression to cervical cancer:

These factors can be multiple full-term pregnancies, early first sexual intercourse, history of STI infection, smoking or breathing in secondhand smoke, long-term use of oral contraceptives, family history of cervical cancer etc.

Cervical cancer is a vaccine-preventable disease.

Early vaccination against HPV is the most effective way to prevent cervical cancer.

HPV疫苗无毒、安全可靠。它模拟了病毒的“外貌”，即蛋白外壳，不含病毒DNA，因此不具备传染性。接种HPV疫苗能有效刺激人体免疫系统产生抗体。

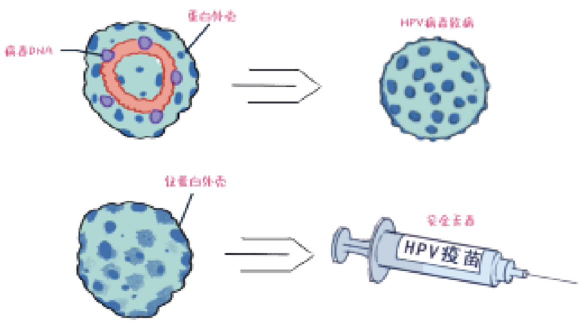

建议9–45岁女性都接种HPV疫苗，预防宫颈癌。一旦感染HPV病毒后，HPV疫苗的保护效果会大大降低，**因此建议尽早接种HPV疫苗，保护效果会更好。**

对比内容	国产二价	进口二价	进口四价	进口九价
接种年龄<br>针剂数量	9–45岁<br>9–14岁接种两针<br>15–45岁接种三针	9–45岁<br>需接种三针	9–45岁<br>需接种三针	16–26岁<br>需接种三针
价格	329元/针<br>全程658/987元	580元/针<br>全程1740元	798元/针<br>全程2394元	1298元/针<br>全程3894元

HPV vaccination is a safe and effective way to help prevent cervical cancer. The current HPV vaccines are based on virus-like particles (VLPs) that are formed by HPV surface components. VLPs are not infectious because they lack the virus’s DNA. The HPV vaccines effectively stimulate the body's immune system to produce antibodies.

The HPV vaccine is recommended for females aged 9 to 45 years to prevent cervical cancer. Once infected with HPV viruses, HPV vaccination has less benefits. It is recommended to get the HPV vaccine as early as possible for better protection.

Comparison Content	Domestic bivalent vaccine (Cecolin)	Imported bivalent vaccine (Cervarix)	Imported quadrivalent vaccine (Gardasil)	Imported 9-valent HPV vaccine (Gardasil 9)
Age for Vaccination and Doses Needed	9-45 years old<br>A 2-dose schedule is recommended for age 9-14 and 3 doses are recommended for age 15-45	9-45 years old<br>A 3-dose schedule is recommended for age 9-45	9-45 years old<br>A 3-dose schedule is recommended for age 9-45	16-26 years old (this age range was recently amended to 9-45 years in August 2022)<br>A 3-dose schedule is recommended for age 9-45
Price	329 yuan/dose (US$49.78 at the exchange rate of US$1=6.61CNY)<br>658/987 yuan altogether(US$99.56/US$149.34)	560 yuan/dose (US$84.72)<br>1740 yuan altogether(US$254.16)	798 yuan/dose (US$120.72)<br>2394 yuan altogether (US$362.18)	1298 yuan/dose (US$196.37)<br>3894 yuan altogether (US$580.11)

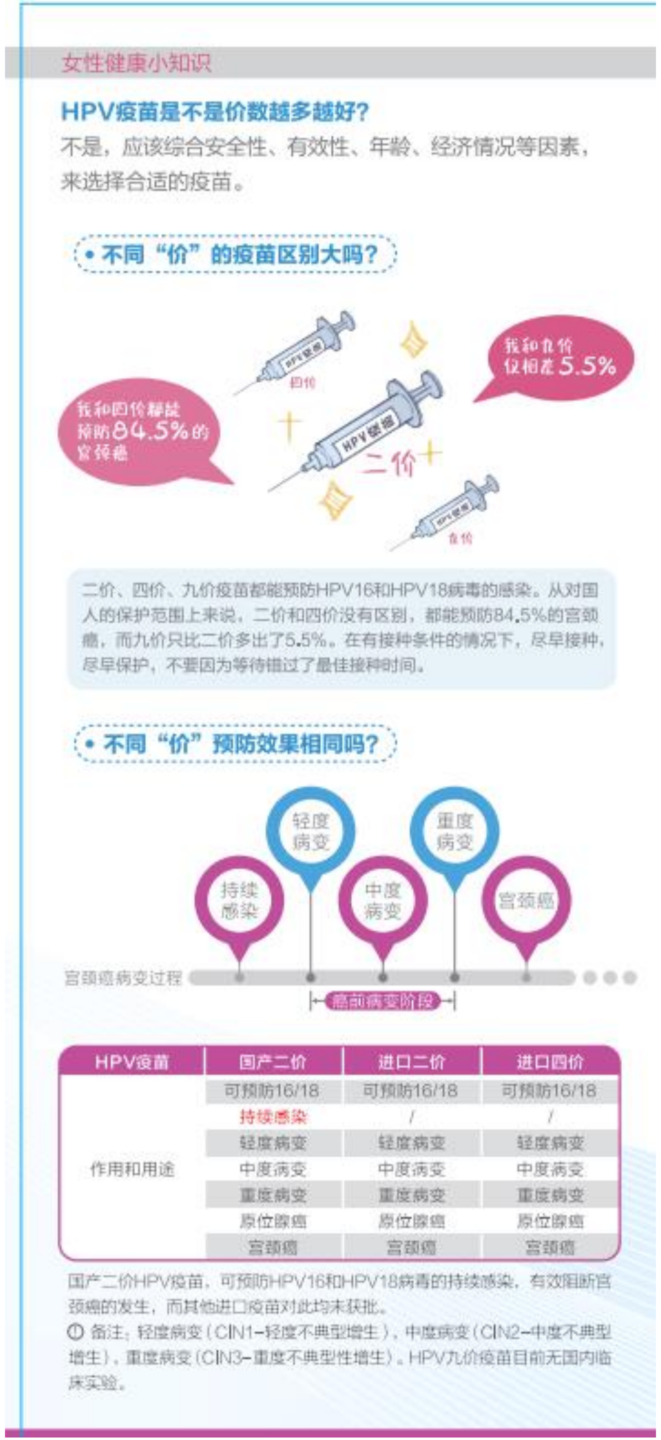

女性健康小知识

HPV疫苗是不是价数越多越好?

不是，应该综合安全性、有效性、年龄、经济情况等因素，来选择合适的疫苗。

• 不同“价”的疫苗区别大吗?

我和四价都能预防84.5%的宫颈癌

我和九价仅相差5.5%

四价

二价

九价

二价、四价、九价疫苗都能预防HPV16和HPV18病毒的感染。从对国人的保护范围上来说，二价和四价没有区别，都能预防84.5%的宫颈癌，而九价只比二价多出了5.5%。在有接种条件的情况下，尽早接种，尽早保护，不要因为等待错过了最佳接种时间。

• 不同“价”预防效果相同吗?

持续感染 轻度病变 中度病变 重度病变 宫颈癌

宫颈癌病变过程

癌前病变阶段

HPV疫苗	国产二价	进口二价	进口四价
作用和用途	可预防16/18	可预防16/18	可预防16/18
	持续感染	/	/
	轻度病变	轻度病变	轻度病变
	中度病变	中度病变	中度病变
	重度病变	重度病变	重度病变
	原位腺癌	原位腺癌	原位腺癌
	宫颈癌	宫颈癌	宫颈癌

国产二价HPV疫苗，可预防HPV16和HPV18病毒的持续感染，有效阻断宫颈癌的发生，而其他进口疫苗对此均未获批。

① 备注：轻度病变（CIN1–轻度不典型增生），中度病变（CIN2–中度不典型增生），重度病变（CIN3–重度不典型性增生）。HPV九价疫苗目前无国内临床实验。

Women's health tips

- **Is the 9-valent HPV vaccine the best?**

No. The choice of HPV vaccine should be based on a number of factors, including safety, efficacy, age, price and so on.

- **Are there any differences between these HPV vaccines?**

Bivalent or quadrivalent HPV vaccines can potentially reduce cervical cancer by 84.5%, which is only 5.5% less than the 9-valent HPV vaccine.

Current evidence suggests that licensed HPV vaccines have relatively similar effectiveness in preventing cervical cancer. With regards to cervical cancer prevention, all licensed HPV vaccines provide high protection against HPV-16 and HPV-18 which are associated with 84.5% of cervical cancer cases in China. It is recommended to vaccinate girls as early as possible, and don't miss the best vaccination time due to waiting.

- **How effective are HPV vaccines?**

HPV vaccine	Domestic bivalent vaccine	Imported bivalent vaccine	Imported quadrivalent vaccines
Functions and Uses	prevent HPV16/18	prevent HPV16/18	prevent HPV16/18
	Persistent HPV infection	/	/
	Mild lesions	Mild lesions	Mild lesions
	Moderate lesions	Moderate lesions	Moderate lesions
	Severe lesions	Severe lesions	Severe lesions
	In situ adenocarcinoma	In situ adenocarcinoma	In situ adenocarcinoma
	cervical cancer	cervical cancer	cervical cancer

The domestic bivalent HPV vaccine can prevent persistent infection of HPV-16 and HPV-18 viruses and effectively prevent cervical cancer.

①Note: mild precancerous lesions(CIN1-mild dysplasia),moderate precancerous lesions(CIN2-moderate to marked dysplasia),severe precancerous lesions(CIN3-severe dysplasia to carcinoma in situ). There are currently no clinical trials for domestic 9-valent HPV vaccines.
