# Supplementary material for: The effectiveness of pay-it-forward in addressing HPV vaccine delay and increasing uptake among 15–18-year-old adolescent girls compared to user-paid vaccination: a study protocol for a two-arm randomized controlled trial in China
Source: BMC Public Health. 2023 Jan 7;23:48. doi: 10.1186/s12889-022-14947-3 (PMC9824916; doi:10.1186/s12889-022-14947-3)
Supplement: Supplementary file 4 — Additional file 4. [file 12889_2022_14947_MOESM4_ESM.docx]

**Pay-it-forward to improve HPV vaccine uptake among 15–18-year-old adolescent girls**

**ID number：**  **ID card number：**

**（PIF group）**

| **Part 1 Basic information** | | | | | | | | | |
| --- | --- | --- | --- | --- | --- | --- | --- | --- | --- |
| 1、Your gender：   1. Male 2. Female   2、Your ethnicity： | | | | | | | | | |
| A、Han | | | | | | | | | |
| B、Other, please specify： | | | | | | | | | |
|  | | | | | | | | | |
| 3、Your date of birth (eg:“1990.01.01) | | | | | | | | | |
| 4、Your current legal marriage status is：   1. Unmarried 2. Engaged or married 3. Separated or divorced 4. Widowed 5. Other, please specify：   5、The relationship between you and your child is：   1. Father 2. Mother 3. Grandfather 4. Grandmother 5. Other, please specify： | | | | | | | | | |
| 6、Your highest educational level is： | | | | | | | | | |
| 1. Primary school or below | | | | | | | | | |
| 1. Junior middle school | | | | | | | | | |
| 1. High school 2. Undergraduate or college 3. Postgraduate | | | | | | | | | |
| 7、Your current occupation is：   1. Civil servant 2. Farmer 3. Ordinary workers (blue collar or labor-intensive work) 4. Company staff (white collar or office work) 5. Technicians 6. Unemployed or retired 7. Other, please specify：   8、Your family income per year：   1. 0-10000 RMB/Year 2. 10000-30000 RMB/Year 3. 30000-80000 RMB/Year 4. 80000-150000 RMB/Year 5. 150000-300000 RMB/Year 6. 300000-1000000 RMB/Year 7. 1000000 RMB/Year or above | | | | | | | | | |
| 9、Have any your family members ever been infected with HPV?   1. Yes 2. No 3. Not sure | | | | | | | | | |
| 10、Have any your family members ever had cervical cancer?   1. Yes 2. No 3. Not sure | | | | | | | | | |
| **Part 2 Vaccination willingness** | | | | | | | | | |
| 11、The researcher has introduced the HPV vaccine and ‘Pay it forward’ program to you. Would you like to participate in the program and let your daughter receive HPV vaccination? | | | | | | | | | |
| 1. Yes | | | | | | | | | |
| 1. No（Please skip to question 13） | | | | | | | | | |
|  | | | | | | | | | |
| 12、If you are willing to have your child get vaccinated, the reasons are **[select all that apply]** | | | | | | | | | |
| 1. It’s already on my list-to-do | | | | | | | | | |
| 1. Protect my child from cervical cancer | | | | | | | | | |
| 1. Friends/family members’ advice | | | | | | | | | |
| 1. Health workers’ advice（irrelevant to the pay-it-forward project） | | | | | | | | | |
| 1. Because of the pay-it-forward project   13、If you are unwilling to have your child get vaccinated, the reasons are (If the answer to question 11 is “yes”, then skip this question) **[select all that apply]**   1. I have insufficient knowledge about cervical cancer and HPV vaccines 2. I am uncertain about the effectiveness of the HPV vaccine 3. My family members are reluctant to have my child get vaccinated 4. Too troublesome 5. I am worried about side effects 6. I am worried about costs 7. Other, please specify：_______ | | | | | | | | | |
|  | | | | | | | | | |
| 14、Would you like to donate some money to support other girls in your community to be vaccinated against HPV？ | | | | | | | | | |
| 1. Yes | | | | | | | | | |
| 1. No | | | | | | | | | |
|  | | | | | | | | | |
| 15、How much would you like to donate to support the next girl？（If the answer to question 14 is "no", then skip this question）   1. 330 RMB（Help the next girl receive one dose of bivalent domestic HPV vaccine） | | | | | | | | | |
| 1. 165 RMB（Help reduce half of the costs of one dose of bivalent domestic HPV vaccine for the next girl） | | | | | | | | | |
| 1. 110 RMB（Help reduce 1/3 of the costs of one dose of bivalent domestic HPV vaccine for the next girl） | | | | | | | | | |
| 1. Other amount, please specify： | | | | | | | | | |
|  | | | | | | | | | |
| 16、What do you think are the benefits of the ‘Pay it forward’ program？**[select all that apply]** | | | | | | | | | |
| 1. Reduce my financial burden | | | | | | | | | |
| 1. I have learned that HPV vaccine can protect my child against cervical cancer | | | | | | | | | |
| 1. More girls can receive HPV vaccination through this program | | | | | | | | | |
| 1. Potentially reduce the spread of HPV infection | | | | | | | | | |
| 1. Spread love and warmth within the community | | | | | | | | | |
| 1. Other, please specify： | | | | | | | | | |
| **Part 3 Vaccine-related information** | | | | | | | | | |
| 17、Have you heard about the HPV vaccination before this intervention？ | | | | | | | | | |
| 1. Yes | | | | | | | | | |
| 1. No | | | | | | | | | |
|  | | | | | | | | | |
| 18、In general, I think HPV vaccination is important (select a level of agreement for each type of HPV vaccine. 2v-HPV vaccines include both imported and domestic vaccines). | | | | | | | | | |
| A Strongly disagree | | B. Disagree | | | C. Agree | | | D. Strongly agree | |
| 9vHPV vaccines: | | | | | | | | | |
| 4vHPV vaccines:  2vHPV vaccines: | | | | | | | | | |
| 19、In general, I think the HPV vaccination is safe. (select a level of agreement for each type of HPV vaccine). | | | | | | | | | |
| A Strongly disagree | | | B. Disagree | | | C. Agree | | | D. Strongly agree |
| 9vHPV vaccines: | | | | | | | | | |
| 4vHPV vaccines:  2vHPV vaccines: | | | | | | | | | |
| 20、In general, I think the HPV vaccination is effective (select a level of agreement for each type of HPV vaccine). | | | | | | | | | |
| A Strongly disagree | B. Disagree | | | C. Agree | | | D. Strongly agree | | |
| 9vHPV vaccines: | | | | | | | | | |
| 4vHPV vaccines:  2vHPV vaccines: | | | | | | | | | |
| 21、Have any of your family members ever been vaccinated against HPV？ | | | | | | | | | |
| 1. Yes | | | | | | | | | |
| 1. No | | | | | | | | | |
|  | | | | | | | | | |
| 22、Have you ever been hesitant about getting the HPV vaccination (except for allergies)? | | | | | | | | | |
| 1. Yes | | | | | | | | | |
| 1. No | | | | | | | | | |
|  | | | | | | | | | |
| 23、Have you ever postponed the HPV vaccination (except for allergies)? | | | | | | | | | |
| 1. Yes | | | | | | | | | |
| 1. No | | | | | | | | | |
|  | | | | | | | | | |
| 24、Have you ever refused to receive the HPV vaccination (except for allergies)? | | | | | | | | | |
| 1. Yes | | | | | | | | | |
| 1. No | | | | | | | | | |
| 25、Have you ever heard about negative information about HPV vaccination? | | | | | | | | | |
| 1. Yes | | | | | | | | | |
| 1. No | | | | | | | | | |
|  | | | | | | | | | |
| 26、Do any of your friends or relatives object to HPV vaccination? | | | | | | | | | |
| 1. Yes | | | | | | | | | |
| 1. No | | | | | | | | | |
|  | | | | | | | | | |
| 27、Have you or people around you had experienced adverse effects associated with HPV vaccination? | | | | | | | | | |
| 1. Yes | | | | | | | | | |
| 1. No | | | | | | | | | |
|  | | | | | | | | | |
| 28、Is the cost a barrier for you or your family members to receive the HPV vaccination? | | | | | | | | | |
| 1. Yes | | | | | | | | | |
| 1. No   29、Do you think that, after HPV vaccination, your child still needs regular cervical cancer screening after they get married/become sexually active?  A、Yes  B、No  30、Which of the above three HPV vaccines would you preferably want your daughter to receive?  A、 2vHPV vaccines (both imported and domestic)， the reason is ________(Please skip to question 33)  B、imported 4vHPV vaccines, the reason is ________  C、imported 9vHPV vaccines, the reason is ________  D、No preferences, all of the above are fine (Please skip to question 33)  E、Not sure (Please skip to question 33)  31、If your community health center has no supply of 4v/9v HPV vaccines, you will let your daughter：  A、 Vaccinate with 2vHPV vaccines (imported or domestic) (Please skip to question 33)  B、Not vaccinate now but wait until 4v/9vHPV vaccines are available in the community  C、Other, ________________(please specify) (Please skip to question 33)  32、How long will you be willing to wait in order for your daughter to receive a preferred type of HPV vaccine?  A. More than five years  B. Two to five years (inclusive)  C. One to two years (inclusive)  D. Six months to one year (inclusive)  E. Six months or below | | | | | | | | | |
| **Part 4 Social relationship**  Please read the following statements about your prosocial behavior and select the response that can best reflect your immediate feelings/reactions. There are no right or wrong answers.  1=Never/Almost Never  2= Rarely  3=Occasionally  4=Often  5=Always/Almost Always  33、I am pleased to help my friends/colleagues in their activities.  34、I share the things that I have with my friends.  35、I try to help others.  36、I am available for volunteer activities to help those who are in need.  37、I am empathic with those who are in need.  38、I help immediately those who are in need.  39、I do what I can to help others avoid getting into trouble.  40、I intensely feel what others feel.  41、I am willing to make my knowledge and abilities available to others.  42、 I try to console those who are sad.  43、I easily lend money or other things.  44、I easily put myself in the shoes of those who are in discomfort.  45、I try to be close to and take care of those who are in need.  46、I easily share with friends any good opportunity that comes to me.  47、I spend time with those friends who feel lonely.    48、I immediately sense my friends’ discomfort even when it is not directly communicated to me. | | | | | | | | | |

**Pay-it-forward to improve HPV vaccine uptake among 15–18-year-old adolescent girls**

**ID number：**  **ID card number：**

**（Standard-of-care group, parents version）**

| **Part 1 Basic information** | | | | | | | | |
| --- | --- | --- | --- | --- | --- | --- | --- | --- |
| 1、Your gender：   1. Male 2. Female   2、Your ethnicity： | | | | | | | | |
| A、Han | | | | | | | | |
| B、Other, please specify： | | | | | | | | |
|  | | | | | | | | |
| 3、Your date of birth (eg:“1990.01.01) | | | | | | | | |
| 4、Your current legal marriage status is：   1. Unmarried 2. Engaged or married 3. Separated or divorced 4. Widowed 5. Other, please specify：   5、The relationship between you and your child is：   1. Father 2. Mother 3. Grandfather 4. Grandmother 5. Other, please specify： | | | | | | | | |
| 6、Your highest educational level is： | | | | | | | | |
| 1. Primary school or below | | | | | | | | |
| 1. Junior middle school | | | | | | | | |
| 1. High school 2. Undergraduate or college 3. Postgraduate | | | | | | | | |
| 7、Your current occupation is：   1. Civil servant 2. Farmer 3. Ordinary workers (blue collar or labor-intensive work) 4. Company staff (white collar or office work) 5. Technicians 6. Unemployed or retired 7. Other, please specify：   8、Your family income per year：   1. 0-10000 RMB/Year 2. 10000-30000 RMB/Year 3. 30000-80000 RMB/Year 4. 80000-150000 RMB/Year 5. 150000-300000 RMB/Year 6. 300000-1000000 RMB/Year 7. 1000000 RMB/Year or above | | | | | | | | |
| 9、Have any your family members ever been infected with HPV?   1. Yes 2. No 3. Not sure | | | | | | | | |
| 10、Have any your family members ever had cervical cancer?   1. Yes 2. No 3. Not sure | | | | | | | | |
| **Part 2 Vaccination willingness** | | | | | | | | |
| 11、The researcher has introduced the HPV vaccine to you. Would you like to participate in the program and let your daughter receive HPV vaccination? | | | | | | | | |
| 1. Yes | | | | | | | | |
| 1. No（Please skip to queation 13） | | | | | | | | |
|  | | | | | | | | |
| 12、If you are willing to have your child get vaccinated, the reasons are **[select all that apply]** | | | | | | | | |
| 1. It’s already on my list-to-do | | | | | | | | |
| 1. Protect my child from cervical cancer | | | | | | | | |
| 1. Friends/family members’ advice | | | | | | | | |
| 1. Health workers’ advice | | | | | | | | |
| 1. Other, please specify：   13、If you are unwilling to have your child get vaccinated, the reasons are (If the answer to question 11 is “yes”, then skip this question) **[select all that apply]**   1. I have insufficient knowledge about cervical cancer and HPV vaccines 2. I am uncertain about the effectiveness of the HPV vaccine 3. My family members are reluctant to have my child get vaccinated 4. Too troublesome 5. I am worried about side effects 6. I am worried about costs 7. Other, please specify：_______ | | | | | | | | |
|  | | | | | | | | |
| **Part 3 Vaccine-related information** | | | | | | | | |
| 14、Have you heard about the HPV vaccination before this intervention？ | | | | | | | | |
| 1. Yes | | | | | | | | |
| 1. No | | | | | | | | |
|  | | | | | | | | |
| 15、In general, I think HPV vaccination is important (select a level of agreement for each type of HPV vaccine. 2v-HPV vaccines include both imported and domestic vaccines). | | | | | | | | |
| A Strongly disagree | B. Disagree | | | | C. Agree | | | D. Strongly agree |
| 9vHPV vaccines: | | | | | | | | |
| 4vHPV vaccines:  2vHPV vaccines: | | | | | | | | |
| 16、In general, I think the HPV vaccination is safe (select a level of agreement for each type of HPV vaccine). | | | | | | | | |
| A Strongly disagree | | | B. Disagree | | | C. Agree | | D. Strongly agree |
| 9vHPV vaccines: | | | | | | | | |
| 4vHPV vaccines:  2vHPV vaccines: | | | | | | | | |
| 17、In general, I think the HPV vaccination is effective (select a level of agreement for each type of HPV vaccine). | | | | | | | | |
| A Strongly disagree | | B. Disagree | | C. Agree | | | D. Strongly agree | |
| 9vHPV vaccines: | | | | | | | | |
| 4vHPV vaccines:  2vHPV vaccines: | | | | | | | | |
| 18、Have any of your family members ever been vaccinated against HPV？ | | | | | | | | |
| 1. Yes | | | | | | | | |
| 1. No | | | | | | | | |
|  | | | | | | | | |
| 19、Have you ever been hesitant about getting the HPV vaccination (except for allergies)? | | | | | | | | |
| 1. Yes | | | | | | | | |
| 1. No | | | | | | | | |
|  | | | | | | | | |
| 20、Have you ever postponed the HPV vaccination (except for allergies)? | | | | | | | | |
| 1. Yes | | | | | | | | |
| 1. No | | | | | | | | |
|  | | | | | | | | |
| 21、Have you ever refused to receive the HPV vaccination (except for allergies)? | | | | | | | | |
| 1. Yes | | | | | | | | |
| 1. No | | | | | | | | |
| 22、Have you ever heard about negative information about HPV vaccination? | | | | | | | | |
| 1. Yes | | | | | | | | |
| 1. No | | | | | | | | |
|  | | | | | | | | |
| 23、Do any of your friends or relatives object to HPV vaccination? | | | | | | | | |
| 1. Yes | | | | | | | | |
| 1. No | | | | | | | | |
|  | | | | | | | | |
| 24、Have you or people around you had experienced adverse effects associated with HPV vaccination? | | | | | | | | |
| 1. Yes | | | | | | | | |
| 1. No | | | | | | | | |
|  | | | | | | | | |
| 25、Is the cost a barrier for you or your family members to receive the HPV vaccination? | | | | | | | | |
| 1. Yes | | | | | | | | |
| 1. No   26、Do you think that, after HPV vaccination, your child still needs regular cervical cancer screening after they get married/become sexually active?  A、Yes  B、No  27、Which of the above three HPV vaccines would you preferably want your daughter to receive?  A、 2vHPV vaccines (both imported and domestic), the reason is ________(Please skip to question 30)  B、imported 4vHPV vaccines, the reason is ________  C、imported 9vHPV vaccines, the reason is ________  D、No preferences, all of the above are fine (Please skip to question 30)  E、Not sure (Please skip to question 30)  28、If your community health center has no supply of 4v/9v HPV vaccines, you will let your daughter：  A、 Vaccinate with 2vHPV vaccines (imported or domestic) (Please skip to question 30)  B、Not vaccinate now but wait until 4v/9vHPV vaccines are available in the community  C、Other, ________________(please specify) (Please skip to question 30)  29、How long will you be willing to wait in order for your daughter to receive a preferred type of HPV vaccine?  A. More than five years  B. Two to five years (inclusive)  C. One to two years (inclusive)  D. Six months to one year (inclusive)  E. Six months or below | | | | | | | | |
| **Part 4 Social relationship**  Please read the following statements about your prosocial behaviors and select the response that can best reflect your immediate feelings/reactions. There are no right or wrong answers.  1=Never/Almost Never  2= Rarely  3=Occasionally  4=Often  5=Always/Almost Always  30、I am pleased to help my friends/colleagues in their activities.  31、I share the things that I have with my friends.  32、I try to help others.  33、I am available for volunteer activities to help those who are in need.  34、I am empathic with those who are in need.  35、I help immediately those who are in need.  36、I do what I can to help others avoid getting into trouble.  37、I intensely feel what others feel.  38、I am willing to make my knowledge and abilities available to others.  39、 I try to console those who are sad.  40、I easily lend money or other things.  41、I easily put myself in the shoes of those who are in discomfort.  42、I try to be close to and take care of those who are in need.  43、I easily share with friends any good opportunity that comes to me.  44、I spend time with those friends who feel lonely.    45、I immediately sense my friends’ discomfort even when it is not directly communicated to me. | | | | | | | | |
